# Manufacturing of Pure Copper with Electron Beam Melting and the Effect of Thermal and Abrasive Post-Processing on Microstructure and Electric Conductivity

**DOI:** 10.3390/ma16010073

**Published:** 2022-12-21

**Authors:** Sandra Megahed, Florian Fischer, Martin Nell, Joy Forsmark, Franco Leonardi, Leyi Zhu, Kay Hameyer, Johannes Henrich Schleifenbaum

**Affiliations:** 1 Digital Additive Production (DAP), RWTH Aachen University, Campus-Boulevard 73, 52074 Aachen, Germany; 2 Institute of Electrical Machines (IEM), RWTH Aachen University, Schinkelstr. 4, 52062 Aachen, Germany; 3 Research and Advanced Engineering Laboratory, Ford Motor Company, 2101 Village Road, Dearborn, MI 48121, USA

**Keywords:** copper, electron beam melting, heat treatment, post-processing, microstructure, electric conductivity

## Abstract

Due to the increasing demand for electrification in the automotive sector, the interest in the manufacturing and processing of pure Copper (Cu; purity 99.99%) is also increasing. Laser-based technologies have proven to be challenging due to Cu’s high optical reflectivity. Processing pure Cu with Electron Beam Melting (EBM) is a promising manufacturing route, allowing for high design freedom. The highest priority is to achieve outstanding thermal and electric conductivity in manufactured Cu components. Chemical contamination or manufacturing defects, such as porosity, significantly reduce the thermal and electric conductivity. The literature on post-processing (thermal and abrasive) of additively manufactured Cu is scarce. Therefore, this study discusses the correlation between as built and heat treated microstructure, as well as surface roughness on the EBM electric conductivity. EBSD analysis is performed to analyze the effect of microstructure on electric conductivity. The effect of sandblasting and vibratory finishing on surface roughness and electric conductivity is investigated. Additionally, the samples are mechanically tested in terms of hardness.

## 1. Introduction

Due to the distinctive combination of physical and mechanical properties, copper and copper alloys are interesting for a wide range of applications [1,2,3]. The high electric conductivity, in particular, allows for its use in electric components, such as winding conductors, motors, and contacts, while the high thermal conductivity makes copper suitable for heat transfer systems, such as radiators and heat exchangers [4,5]. Thus, there is a strong interest in the industrial production of complex copper shapes in automotive applications.

Powder bed fusion (PBF) systems are commonly used to print 3D models by using either a laser beam (L-PBF) or an electron beam (electron beam melting, EBM) as an energy source [6,7]. Fundamentally, these systems work alike: metal powder is deposited on a build platform and the energy source melts a layer of the deposited powder according to the corresponding sliced geometry. The build platform is lowered, and the process is repeated until the complete part is manufactured. 

While pure copper reflects up to 98% of infrared laser radiation commonly used in L-PBF [8,9], requiring a high level of energy to produce a melt pool, pure copper actually absorbs 80% of electron beam energy, thus making the EBM process much more flexible [10,11]. The processability of Cu with EBM has been validated in previous studies [3,12,13], with relative densities higher than 99.5% being reported [14]. Microstructural analysis shows that EBM-printed pure copper samples predominantly form long columnar grain structures parallel to the build direction [13].

Hardness values of 46–48 HV and tensile strengths of up to 231.6 MPa with an elongation at fracture of 59.3% were achieved with EBM [11,15]. The tensile strength and Young’s modulus are greatly influenced by the grain orientation. The tensile strength is highest when the load is applied perpendicular to the columnar grains and is lowest when applied parallel to the grain orientation. The same principle applies to Young’s modulus, where a maximum of 145.5 GPa is achieved. For samples with columnar microstructure, grain orientation in <001> and <101> directions are predominant [11].

Guschlbauer et al. [11] studied the effect of oxygen content on part performance during the EBM process. A high oxygen content (~0.2 wt.%) in the powder led to increased crack formation and therefore inferior mechanical properties, while samples manufactured from powder with low oxygen content achieve properties similar to typical wrought copper. 

Research work on typical post-processing methods for EBM parts, to further tailor the microstructure of pure copper, is limited. Studies with conventionally manufactured copper alloys show great improvements in mechanical and physical properties with conventional two-step heat treatments. Since an in situ heat treatment is incorporated during the EBM process [16], thermal post-processing is often not carried out. The effect of heat treatment on EBM Cu on microstructure and mechanical properties is, therefore, not fully known.

Recently, the physical properties of EBM-manufactured copper have been examined [17]. Jiang et al. summarized the reported electric conductivities alongside the used EBM process parameters [17]. An Area Energy Density (E_A_) around 5 J/mm^2^ achieved the highest electric conductivities; >99.6–100% International Annealed Copper Standard (IACS). Electric conductivities of 94%IACS were achieved with an E_A_ of 11 J/mm^2^ [11,12,13,14,15]. 

Using the Wiedemann–Franz Law (refer to [18] for further details), an electric conductivity of 55.9 MS/m^−1^ is expected for ideal conditions (i.e., no defects, no impurities etc.). A 100 %IACS is equivalent to 59.7 MS/m^−1^, meaning that the expected electric conductivity lies at 94%IACS. This would agree with the results found by Raab et al. [14], who related the deviation in electric conductivity from 100%IACS to raw materials characteristics and chemical impurities. 

Since the effect of microstructural characteristics on EBM electric conductivity is largely unexplored, this study focuses on the EBM manufacture of pure copper samples, the microstructure in an as built and heat treated condition, and the correlation with the resulting electric conductivity. Special focus is also placed on surface roughness, since the extent of different types of abrasive post-processing on improving surface quality is unknown. The correlation of electric conductivity and surface roughness is also unexplored for EBM Cu samples in the literature, and has been carried out in this study. 

## 2. Materials and Methods

### 2.1. Copper

Pure copper powder (purity 99.98%) was supplied by Eckart TLS GmbH (Bitterfeld-Wolfen, Germany). The powder was gas atomized under argon atmosphere. The powder showed an average sphericity of 0.85 (measured with ImageJ open-source software) and showed little to no porosity. Limited satellites could be identified. D_10_, D_50_ and D_90_ were 50.97 µm, 68.81 µm and 88.99 µm, respectively. Powder analysis showed an oxygen content of a maximum of 0.02%. 

### 2.2. Electron Beam Melting

Cubes (10 mm × 10 mm × 20 mm) were built on an Arcam Q10plus machine (ArcamEBM: A GE Additive company, Gothenburg, Sweden). Key EBM parameters that were varied within the scope of this study were line offset (0.1–0.2 µm) and the scan speed (600–6000 mm/s). With these parameters, the Area Energy Density (E_A_ = $\frac{\mathrm{Beam}\;\mathrm{power}}{\mathrm{Line}\;\mathrm{offset}\;\times \;\mathrm{Scan}\;\mathrm{speed}}$) could be varied between 2–9 J/mm^2^. The layer thickness was 50 μm. The samples were manufactured on a Cu substrate plate with a preheating temperature of 350 °C. 

### 2.3. Surface Roughness, Relative Density, Microstructure and Vickers Hardness

The surface roughness of cubes was measured using a Keyence VHX7000 (Keyence Germany GmbH, Neu-Isenburg, Germany). By analyzing the depth of focus, a 3D model of the surface was obtained on which surface roughness can be measured. 

For relative density analysis, samples were cold mounted in epoxy resin, ground up to 4000 grade sandpaper and subsequently polished manually with a 1 µm diamond suspension. Relative density was measured using the Keyence VHX7000. Using 100× magnification, images of the entire surface were stitched together. The built-in Keyence software distinguished pores and solid material based on contrast changes.

For the analysis of the microstructure, samples were etched with 10% ferric nitrate solution. A Zeiss Scanning Electron Microscope (SEM; Carl Zeiss AG, Oberkochen, Germany) was used. An EDX spectrum was measured to verify chemical composition. EBSD analysis was carried out for a 1 mm × 3 mm field. Average grain size, grain orientation and grain boundary angles were calculated. 

Using the same samples as for the relative density measurements, the hardness (HV0.2) was measured with the Qness 30A+ microhardness tester (ATM Qness GmbH, Mammelzen, Germany) along the build direction, in the middle of the sample. A mean of 3 measurements per location was calculated. 

### 2.4. Thermal and Abrasive Post-Processing

Three heat treatments were chosen and are shown in Table 1. All heat treatments were carried out under argon atmosphere with a gas flow of 250 nl/h. The heating rate was set at 5 °C/min. 

To improve the surface roughness, the test geometries were sand blasted and underwent vibratory finishing. The post-processing methods are listed in Table 2.

### 2.5. Electric Conductivity

The electric conductivity was measured parallel to the grain boundaries for as built, sand blasted and heat treated samples. The sample geometry was 3 × 3 × 62 mm^3^ (L × B × H) with larger gripping areas at each end. The four-terminal sensing method was applied. The distance between the voltage measurement points was 59 mm. An average was calculated for 5 samples for each sample condition.

## 3. Results and Discussion

### 3.1. EBM Process Window Development

**Figure 1:**
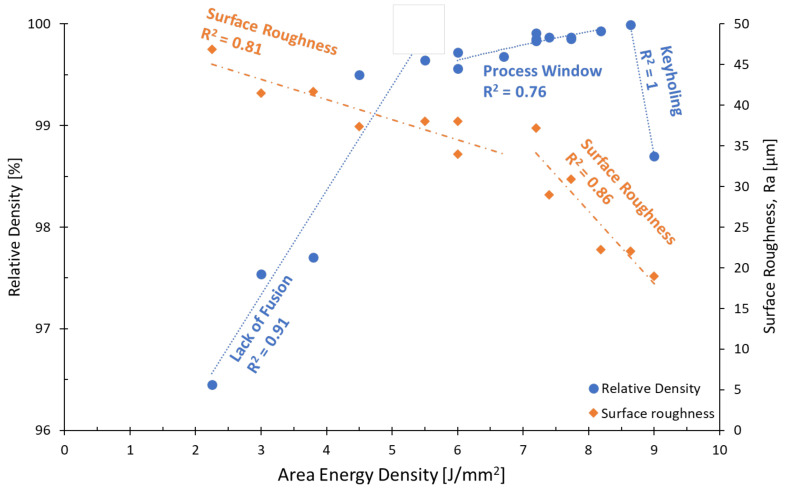
Relative Density and Surface Roughness vs. Area Energy Density of EBM Cu samples.

A total of five build jobs were performed. Each build job included nine cubes. Each cube was printed with a different set of process parameters, where the E_A_ was varied between 2 and 9 J/mm^2^. The relative density and surface roughness are shown for the studied E_A_ range in Figure 1. Since defects, such as porosity, significantly reduce the electric and thermal conductivity and mechanical properties, due to dislocation hindrance, residual stresses, stress concentrations etc., the aim was to develop a process window to achieve a relative density of 99.99%. 

**Figure 2:**
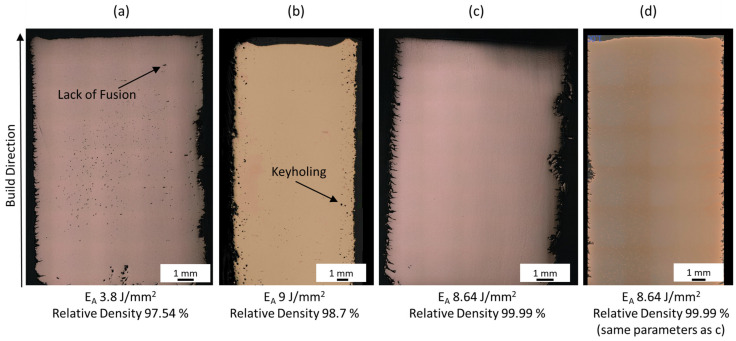
(**a**) Cube with Lack of Fusion; (**b**) Cube with Keyholing; (**c**) Cube with relative density of 99.99%; (**d**) Cube with the same parameters as in (**c**) to verify reproducibility.

The relative density in Figure 1 shows three regions with distinct characteristics: The first is in the range below 6 J/mm^2^, where there is not enough energy to fuse and melt the powder leading to lack of fusion porosity. The pores in this region are known to be irregular in shape, as can be seen in Figure 2a. The second region, between 6 and 8.8 J/mm^2^ is the most relevant region, where the highest relative densities are achieved. The Cu relative density increases with increasing energy density up to 8.8 J/mm^2^. However, a drop in the gradient can be seen at 6 J/mm^2^. In the third region (above 8.8 J/mm^2^) too much energy density is delivered to the powder bed, leading to keyholing and an increase in gas enclosures, typically spherical in shape (see Figure 2b). The relative density results in this study are within the bounds compiled by Ledford et al. [19], who summarized Cu EBM processing windows found in the literature. 

The regions of interest are best correlated with three linear regression curves with R^2^ scores between 0.76 and 0.91. The regression curve in the keyholing region joins two data points only, and is thus statistically questionable. The R^2^ score of the second region might be improved by more data points. Nevertheless, the lowest level of porosity is achieved at E_A_ 8.64 J/mm^2^, with which a maximum relative density of 99.99% was achieved. Figure 2c shows the corresponding cross-section with no visible defects at all. The difference in E_A_ between Figure 2b,c is less than 0.5 J/mm^2^, indicating that the process window of pure Cu is small and sensitive to changes in process parameters. Figure 2d shows a cube that was printed in a separate build job, to verify the reproducibility of the identified process parameters. During the second set of experiments, the powder was recycled and sieved twice before reuse. The roughness on the cube sides corresponds to the engraved cube numbers. 

An energy density of 8.64 J/mm^2^ showed reproducible relative densities of 99.99% without defects, such as porosity. All experiments regarding electric conductivity were carried out with an E_A_ of 8.64 J/mm^2^. Therefore, porosity can be ruled out as possible reasonings for the reported material behavior. 

A similar trend line curve for relative density, as shown in Figure 1, was found by Jiang et al. [17] and Dadbakhsh et al. [20]. While the energy density threshold depends on the material to be processed, the three sections (Lack of Fusion, Process Window and Keyholing) and their trend with energy density are commonly found for all materials and AM technologies [21,22,23,24,25].

Figure 1 also shows that the surface roughness is inversely proportional to the energy density. The surface roughness is best correlated with two linear regression curves joined at 7 J/mm^2^. The R^2^ score of both regressions is higher than 0.81. A minimum surface roughness of 19 µm at 9 J/mm^2^ was measured corresponding to a relative density of 98.7 %. At lower energy densities, particles sinter/partially melt onto the sample surface. With increasing energy density, particles fully melt and do not retain the spherical shape, thereby reducing the surface roughness. The minimum surface roughness is thus obtained using an energy density that is outside of the recommended range for maximum relative density (between 6 and 8.8 J/mm^2^). A compromise can thus be found at E_A_ 8.64 J/mm^2^, yielding 99.99% dense cubes and a surface roughness of 22 µm.

### 3.2. As Built Microstructure and Electric Conductivity

EBSD analysis was carried out for four samples manufactured with E_A_ 4.5 J/mm^2^, 7.57 J/mm^2^, 7.7 J/mm^2^ and 8.64 J/mm^2^. The results are compared in Table 3. The microstructures for all energy densities show columnar grains oriented in the build direction. This is in agreement with the literature [11,13,26]. For 4.5 J/mm^2^, 7.57 J/mm^2^, and 8.64 J/mm^2^, no other orientation besides [001] is present. This can be attributed to grains growing along the thermal gradient, parallel to the build direction. 

For the samples manufactured with energy densities 7.57 J/mm^2^ and 7.7 J/mm^2^, the differences in microstructure are considered to be negligible. Therefore, the EBSD analysis of 7.7 J/mm^2^ is rotated by 90° to identify changes in grain orientation, parallel to the build direction. While more grain orientations are recorded, columnar grains can still be seen. The epitaxial grain growth can clearly be identified in the 7.7 J/mm^2^ EBSD analysis, since some grain lengths exceed 3 mm, corresponding to more than 60 layers. The grains with unfavorable grain orientation are overgrown by grains oriented in the build direction.

The average grain sizes increase with the energy densities, as shown in Table 3. For the highest relative density at E_A_ 8.64 J/mm^2^, the grain size is 55.3 µm. The heat input, combined with the elevated preheat temperature throughout the build (i.e., 350 °C), leads to grain coarsening. The effect of in situ heat treatment during EBM has previously been reported [16,27]. The grain size of the 7.7 J/mm^2^ sample does not follow the increasing grain size with energy density trend. This is due to the orientation of the EBSD scan. Along the build direction, grains with unfavorable grain orientation are overgrown and are hence smaller compared to those with favorable grain orientation. The average grain size of the 7.7 J/mm^2^ sample is, therefore, not representable, since smaller overgrown grains are also considered. The grain orientation is not affected by the energy density in the investigated energy density range. 

**Figure 3:**
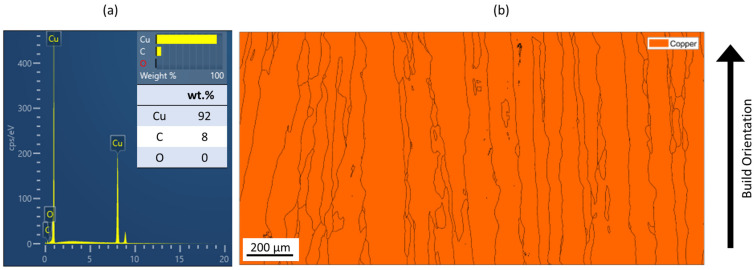
(**a**) EDX spectrum of Cu Sample manufactured with 8.64 J/mm^2^; (**b**) EBSD Phase Analysis of Cu sample with E_A_ 8.64 J/mm^2^.

Figure 3a shows the EDX spectrum for the samples printed with 8.64 J/mm^2^ (relative density 99.99%). Five EDX spectra were taken, and since all spectra are similar to each other, one representative spectrum was chosen. As can be seen from Figure 3a, mostly Cu was detected. Up to 8 wt.% carbon is shown. Carbon with an atomic number below 11 can be detected with EDX, however, it cannot be accurately quantified. The epoxy resin, in which the Cu samples were embedded, contains carbon fibers to ensure electric conductivity within the SEM. The detected carbon is therefore from the epoxy resin, rather than from the Cu samples themselves. Oxygen was not measured. No precipitations formed that could have affected the mechanical or electric properties. The EBSD-phase analysis in Figure 3b further confirms that no impurity phases formed at the measured scale that could negatively affect electric conductivity. All grains show the same phase. Similar results are found for samples with E_A_ 4.5 J/mm^2^, 7.57 J/mm^2^, 7.7 J/mm^2^ and 8.64 J/mm^2^.

**Figure 4:**
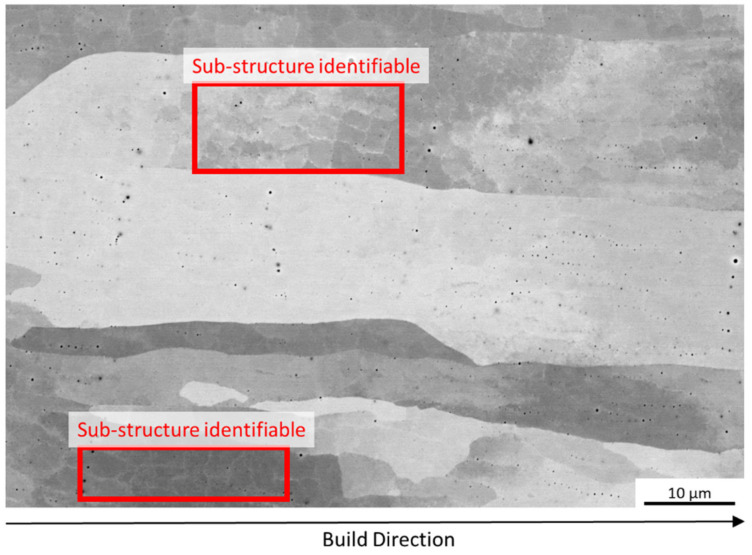
SEM Image of microstructure of cube (E_A_ 4.5 J/mm^2^) with relative density >99.5 %, sub-grain structure can clearly be identified.

An SEM-image for a cube manufactured with E_A_ 4.5 J/mm^2^ (relative density > 99.5 %) is shown in Figure 4. Oriented grains similar to those shown in Table 3 with some small pores can be seen. A grain sub-structure can be identified, which is similar to that reported by Ramirez et al. [28]. The sub-structure was attributed to Cu_2_O, which was measured at a finer scale than used in this study. The sub-structure shown in Figure 4 is possibly due to the powder oxygen content (see powder characterization above) or additional oxygen uptake during the recycling process that could not be resolved using EDX analysis. TEM analysis is necessary to confirm the presence of Cu_2_O. The sub-structure was also found for higher energy densities.

Based on the compilation of Jiang et al. [17], it can be assumed that the electric conductivity of the manufactured samples in this study lies between 94–100 %IACS. The measured electric conductivity for an as built sample parallel to the build direction and grain orientation (as shown in Table 3) at E_A_ 8.64 J/mm^2^ is 39.8 MSm^−1^ ± 2.1 MSm^−1^. This is equivalent to 66.66%IACS, which is significantly lower than expected. 

**Figure 5:**
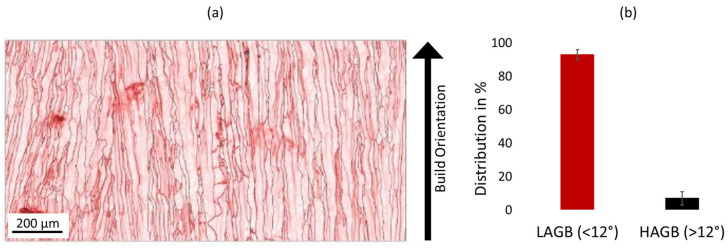
(**a**) EBSD Grain boundary angles of Cu sample with E_A_ 8.64 J/mm^2^ (High angle grain boundaries = Black lines, >10°; Low angle grain boundaries = Red lines, <10°); (**b**) Distribution of LAGBs and HAGBs from EBSD scan shown in (**a**).

Figure 5a shows the grain boundaries for an E_A_ 8.64 J/mm^2^ sample. The black lines indicate angles greater than 10° (i.e., high angle grain boundaries—HAGB), and red lines show grain boundary angles below 10° (i.e., low angle grain boundaries—LAGB). Figure 5b shows the distribution of LAGBs and HAGBs in the EBSD scan of Figure 5a. It can be clearly identified that the amount of LAGBs dominates, compared to the limited number of HAGBs. Dislocation movement is facilitated in LAGBs, since LAGBs ensure coherency within the atom structure of the material. However, dislocations, similar to grain boundaries, act as a hindrance to electric conductivity. LAGBs, therefore, reduce electric conductivity due to increased dislocation mobility. Some grain boundaries show curvature, which can reduce the electric conductivity by up to 80% [29]. Straight grain boundaries are, therefore, preferred and can be achieved by increasing the overall build temperature, thereby increasing the thermal gradient. 

The reduced electric conductivity measured (i.e., 38.9 MSm^−1^) is attributed to the amount and curvature of LAGBs. The possible presence of Cu_2_O precipitates in the sub-structure further reduces the electric conductivity. 

### 3.3. Abrasive Post-Processing and Electric Conductivity

**Figure 6:**
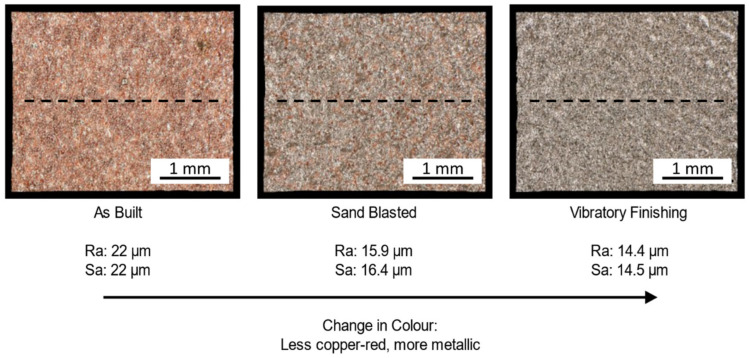
Post-processed Cu samples and the respective surface roughness. The dashed line indicates the line scans.

Figure 6 shows the effect of abrasive post-processing on the surface roughness of as built Cu samples. The surface roughness (R_a_ values) is reduced, compared to the as built condition, by up to 35%. The area surface roughness (S_a_) shows similar results. Sand blasting and vibratory finishing remove sintered and partially molten particles from the sample surface, reducing the surface roughness. Compared to sand blasting, vibratory finishing improved the surface roughness by 7%. This can be attributed to the more aggressive collision of the polymer granulates on the surface, as well as the high frequency applied during vibratory finishing. The duration of the vibratory finishing process is also three times longer compared to sand blasting. However, all cubes were post-processed simultaneously during vibratory finishing, whereas each sample was individually sandblasted. The time efficiency of vibratory finishing should also be considered. 

A decrease in surface roughness led to an improved electric conductivity, as seen in the measured electric conductivity after sand blasting in this study. The electric conductivity increased from 38.9 MSm^−1^ (as built condition) to 44.72 MSm^−1^ ± 6.29 MSm^−1^ (as built and abrasively post-processed). This corresponds to an increase of 12% compared to the as built condition. 

With an increased surface roughness, the overall thickness of the sample does not remain constant. The electric conductivity is, therefore, hindered at the edges of the samples increasing the electric impedance. Reducing the surface roughness ensures a more constant sample thickness and that electric conductivity can flow through the sample with reduced impedance. 

### 3.4. Heat Treated Microstructure and Electric Conductivity

**Figure 7:**
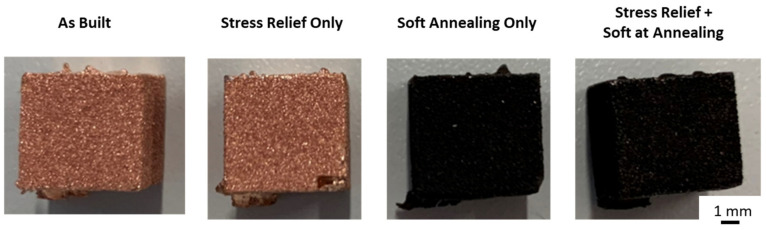
Heat treated Cu samples.

Figure 7 shows heat treated samples. The heat treatments used are listed in Table 1. With increasing heat treatment temperatures and durations, the samples darken, indicating oxidation. It is assumed that there was still residual oxygen left within the oven atmosphere, leading to the observed oxidation. The relative density is unaffected by the heat treatment; all heat treated samples were printed with 8.64 J/mm^2^, and have a relative density of 99.99%. The surface roughness was not affected by heat treatment. 

**Figure 8:**
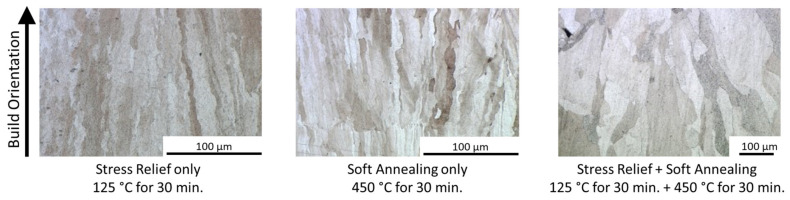
Microstructure of heat treated samples, the arrow indicates the build direction.

**Figure 9:**
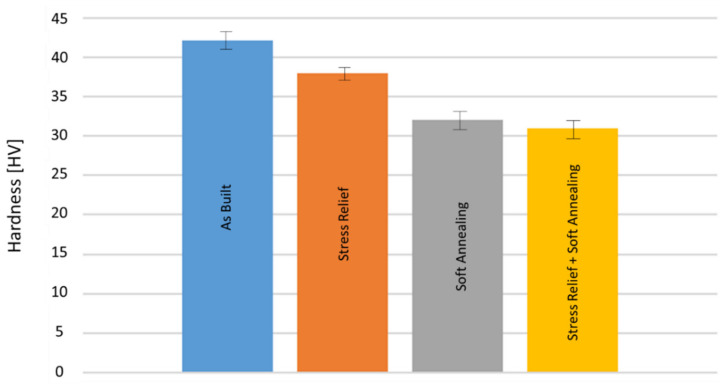
Vickers Hardness of as built and heat treated Cu samples.

The microstructures of the heat treated samples are shown in Figure 8. As can be seen, the grain sizes are larger compared to those shown in Table 3 and increase with increasing heat treatment temperatures, due to grain coarsening. However, the grains do remain oriented in the build direction. Accordingly, Vickers hardness decreases with increased heat treatment temperatures and durations (see Figure 9), according to the Hall–Petch Relation, which states that larger grain sizes lead to reduced hardness. The maximum hardness was measured in the as built condition with 42 HV. The lowest hardness was 31 HV in stress relieved and soft annealed samples. For a higher hardness, a fine and equiaxed microstructure is required. In order to achieve an equiaxed microstructure, a hardening step, with subsequent quenching causing recrystallization during heat treatment, should be considered in future studies. 

**Figure 10:**
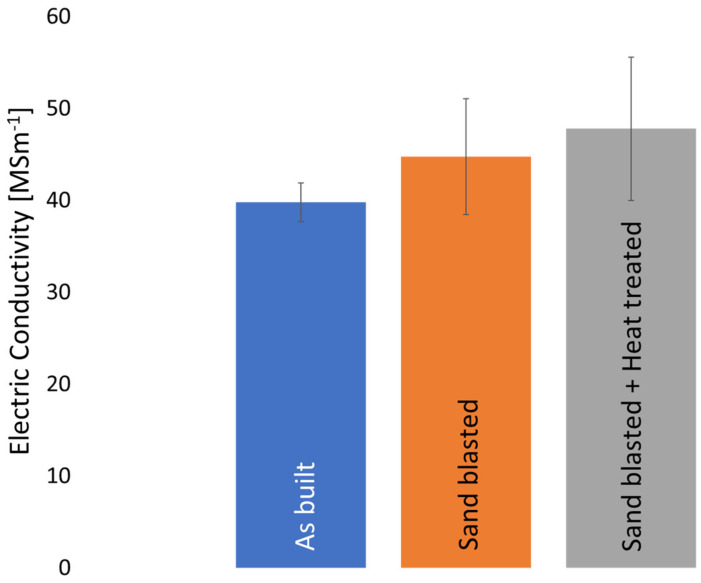
Measured electric conductivity of this study.

After heat treatment (soft annealing), the electric conductivity rises from 44.72 MSm^−1^ (as built and sandblasted) to 47.46 MSm^−1^ ± 7.77 MSm^−1^ (refer to Figure 10), which is an increase of 6%. As previously discussed, grain boundaries reduce electric conductivity. An increase in grain size reduces the overall number of grain boundaries, thereby increasing electric conductivity. This indicates a trade-off between mechanical properties (i.e., hardness) and electric conductivity. Electric conductivity requires a large grain size, whereas a small grain size is required for high hardness. While the focus of this study lies on electric conductivity, it is expected that thermal conductivity is significantly affected by similar factors, i.e., defects, grain boundary angles and grain size. Since pure Cu was investigated and a 99.99% relative density was achieved, it is expected that the thermal conductivity can be calculated using CALPHAD methods. 

Even though an increase in electric conductivity was found, with a decrease in surface roughness and heat treatment (see Figure 10) in this study, the electric conductivity obtained is still lower than expected. It was found that grain boundary angle and curvature are directly corelated to electric conductivity. Heat treatment and improving surface roughness are means of improving electric conductivity, however, the correlation to electric conductivity is not as strong as that of microstructure. 

## 4. Conclusions and Outlook

In this study, the correlation between as built and heat treated microstructure, as well as surface roughness on the EBM electric conductivity of pure copper, is investigated. The following conclusions can be drawn:

- Electric conductivity is heavily dependent on grain boundary angles, chemical composition and surface finish.
- An increase in electric conductivity is achieved by increasing grain size. Further research into achieving a single crystal with EBM should be considered to study the effect on electric conductivity.
- Straight grain boundaries improve electric conductivity. This can be achieved by increasing the overall build temperature.
- There is trade-off between hardness and electric conductivity: For high electric conductivity, large grains are required. For high hardness, a small grain size is needed.
- Chemical impurities, at smaller scales than EDX or EBSD, lead to a sub-structure within grains and significantly reduce electric conductivity.

## Figures and Tables

**Table 1:** Heat treatments of EBM Cu samples.

As Built	Stress Relief	Soft Annealing	Stress Relief + Soft Annealing
Samples investigated after the EBM process (no further heat treatment)	125 °C for 30 min.	450 °C for 30 min.	125 °C for 30 min. + 450 °C for 30 min.

**Table 2:** Post-processing methods applied in this study.

As Built	Sand Blasting	Vibratory Finishing
Samples investigated after the EBM process (no further surface treatment)	Machine: MHG SMG50 (MHG Strahlanlagen GmbH, Düsseldorf, Germany) 10 min 4 mbar Glass beads ◦ 100–200 µm	Machine: Rösler D150 (Rösler Oberflächentechnik, GmbH, Untermerzbach, Germany) 30 min Program for soft materials Up to 1000 RPM at 50 Hz Cone shaped polymer granulates

**Table 3:** Overview EBSD Analysis for different Area Energy Density.

4.5 J/mm^2^	7.57 J/mm^2^	7.7 J/mm^2^	8.64 J/mm^2^
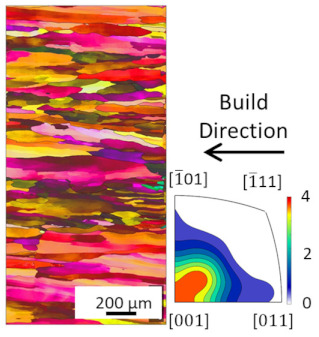	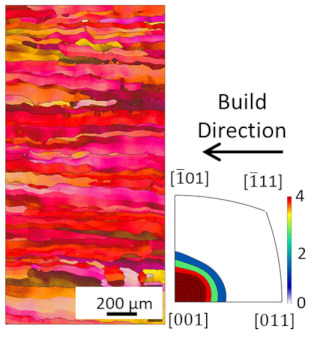	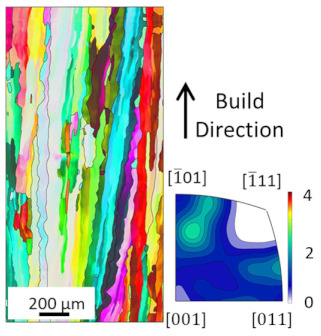	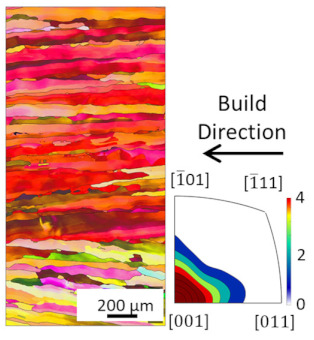
>99.5%Relative Density	>99.5%Relative Density	>99. 5%Relative Density	99.99%Relative Density
30.41 µm Average Grain Size	38.92 µm Average Grain Size	37.73 µm Average Grain Size	55.3 µm Average Grain Size

## Data Availability

Data is not available.
